# In Vivo Animal Study of a Highly Viscous N-butyl Cyanoacrylate Medical Adhesive for Intravenous Embolization

**DOI:** 10.3390/ma14133527

**Published:** 2021-06-24

**Authors:** Jae-Won Seo, Habeen Park, Dogeun Kim, Seoyun Lee, Young Gook Koh, Jang Yong Kim, Insoo Park, Wonmok Lee

**Affiliations:** 1 ENGAIN Co. Ltd., 700, Daewangpangyo-ro, Bundang-gu, Seongnam 13488, Gyeonggi-do, Korea; jwseo@engain.co.kr (J.-W.S.); clooney.park@engain.co.kr (H.P.); dgkim@engain.co.kr (D.K.); sylee@engain.co.kr (S.L.); young.koh@engain.co.kr (Y.G.K.); 2 Department of Chemistry, Sejong University, 209, Neungdong-ro, Gwangjin-gu, Seoul 05006, Korea; 3 Department of Surgery, Seoul St. Mary’s Hospital, College of Medicine, The Catholic University of Korea, Seoul 06591, Korea; vascularkim@catholic.ac.kr; 4 Charm Vascular Clinic, 1814 Nambu-sunhwan-ro, Gwanak-gu, Seoul 08787, Korea; insoo.park.md@gmail.com

**Keywords:** N-butyl cyanoacrylate, medical adhesive, high viscosity, vascular occlusion, embolic agent, heat of polymerization, animal test

## Abstract

N-butyl cyanoacrylate (NBCA) is a liquid monomer that undergoes an exothermic polymerization reaction to form a solid upon initiation with hydroxyl anions. Recently, EGpresto, a highly viscous NBCA-based adhesive, has been developed for vascular-occlusion purposes. In this study, we investigated the heat of polymerization of EGpresto and compared the results with those of a low-viscosity NBCA glue. Results show that EGpresto exhibited a lower heat of polymerization (64 ± 7 °C vs. 34 ± 1 °C). This was due to its high viscosity, which resulted in a delayed polymerization time. To investigate the efficacy and safety of EGpresto for intravenous embolization, a 14 d in vivo animal test was conducted using three pigs. Five cc of EGpresto was injected into the epigastric vein of each animal. Complete postoperative vein occlusion was confirmed at 7 and 14 d by ultrasonographic visualization. After the animals were sacrificed, the operated and unoperated veins were exposed, and the injected adhesive was found without migration. During the histology, the injected adhesive was not found in the inner or outer vein walls, and the immune reactions seemed to be the only foreign-body reaction, showing that EGpresto is a non-toxic and safe intravascular embolic agent.

## 1. Introduction

The clinical use of N-butyl cyanoacrylate (NBCA)-based medical adhesives has been rapidly increasing in recent years, particularly for use as embolic agents for endovascular treatments such as transcatheter arterial embolization [1,2,3], arteriovenous malformations [4,5,6,7], and venous occlusion [8,9,10]. NBCA is a liquid acrylic monomer, and its polymerization reaction can be initiated in an aqueous environment. Because it is rapidly cured into a solid form under intervascular conditions, NBCA adhesive is an appropriate choice for immediate occlusion of the artery and vein [11]. In the human body, the polymerization time of the NBCA adhesive is too fast (typically less than 1 min). Thus, a mixture of NBCA and an oily diluent such as Lipiodol^®^ (Guerbet, Villepinte, France), which is a hydrophobic liquid contrast agent, has been used for retarded embolization [12,13,14]. The other disadvantage of NBCA is its high heat of polymerization [15], which often results in a local temperature increase of over 70 °C during application. This type of heat generation can result in critical side effects such as thermal burns at the treatment site [16].

Histoacryl^®^ (B. Braun Melsungen AG, Melsungen, Germany) is the most common NBCA-based medical adhesive used as an embolic agent. However, Histoacryl was originally approved for topical skin adhesives but not legally approved for use as an embolic agent. Nonetheless, embolization with Histoacryl is allowed for cases of “off-label indication” [17,18]. Recently, we reported a comparison of Histoacryl and EGglue^®^ (ENGAIN, Seongnam, Korea) as embolic materials, and we found that the heat of polymerization and polymerization for the two glues were comparable. However, both glues exhibited a rapid polymerization time (less than 1 min), and thus the control of injection time was found to be difficult [19]. When NBCA is processed as a liquid embolic agent, it is usually mixed with ethiodized oil as a diluent before operation [20]. When it is inserted into a vessel in vivo, the NBCA material ends up being a porous structure upon removal of the diluent in the blood vessel, which then tends to break and migrate to other sites [21]. In a case report, low viscosity of NBCA was found to be a reason for complications such as ischemic pulmonary embolism [18].

**Figure 1:**
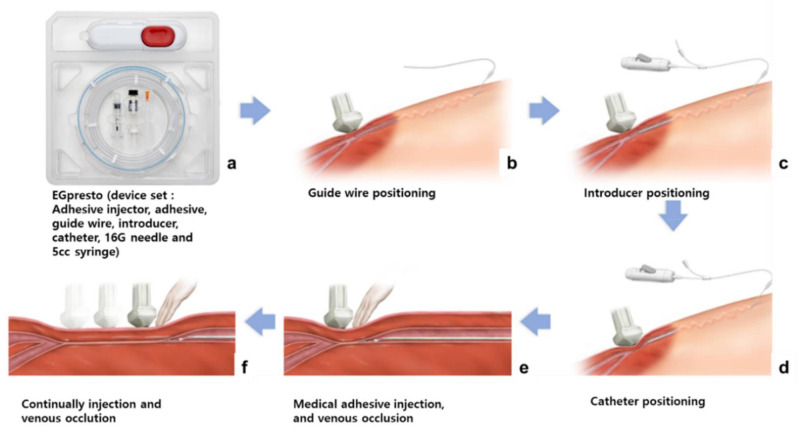
Schematic of the procedures for EGpresto™, a new venous occlusion system. (**a**) EGpresto comes in a plastic pouch containing a highly viscous glue in a vial, along with an injector, guide wire, introducer, catheter, 16 G needle, and a 5 cc syringe. (**b**) In the procedure, the guide wire is introduced through a small scission at the puncture site of the blood vessel. (**c**) The introducer is positioned. (**d**) The catheter is positioned at the location where the first glue will be discharged. (**e**) The adhesive is injected and pressed until the glue is cured. (**f**) After the catheter is pooled several cm back, the adhesive is injected again. The procedure is repeated until the venous occlusion is completed.

Recently, EGpresto™ (ENGAIN, Seongnam, Korea), a highly viscous medical adhesive, has been developed as a new endovenous closure device (Figure 1 and Figure S1). EGpresto was designed to have an optimized formulation that includes NBCA and an organic polymer-based thickener to achieve an optimal viscosity and thus a delayed polymerization time for intravascular occlusion.

The objective of this study is to investigate the physical properties of a new adhesive by comparing it with a low-viscosity medical adhesive already on the market. In addition, we investigate the embolization effect of the new adhesive using an in vivo test with a swine model over 14 d.

## 2. Materials and Methods

### 2.1. Viscosity

The viscosities of the two NBCA glues (EGpresto and EGglue) were determined using a cone-plate viscometer (DV2T; Brookfield, Middleboro, MA, USA) with varying spindle velocity under a controlled temperature of 25 °C. RheocalcT software (version 1.2.19, Brookfield, Middleboro, MA, USA) was used to analyze the measured viscosity data.

### 2.2. Heat of Polymerization and Curing Time

Once initiated by trace amounts of hydroxyl anions, NBCA undergoes an exothermic reaction during polymerization. Experimentally, the curing time is defined as the time required to reach the maximum temperature during polymerization [22]. 

The heat of polymerization and the curing time of the two medical adhesives were determined using an infrared-traceable thermometer gun (Traceable Products, Webster, TX, USA) consisting of a wire-type temperature probe and digital timer. In general, polymerization occurs as soon as an NBCA adhesive is exposed to an aqueous environment. Twenty microliters of cell culture medium (Dulbecco’s modified Eagle’s medium-high glucose, Sigma Life Science, Haverhill, UK) was placed in a 1.5 mL polypropylene tube into which 50 μL of EGpresto or EGglue was subsequently dropped. The change in the solution’s temperature was monitored for 3 min. 

### 2.3. Animal Study Design

All animal study procedures were performed under general anesthesia and monitored by a veterinarian. Three male mini-pigs (Optipharm, Cheongju, Korea) weighing 70–80 kg at 23–24 months of age were used. All three pigs were sacrificed 14 d after EGpresto injection. They were housed in separate cages with the light turned on/off in cycles of 12 h. Illuminance was maintained at 150–300 Lx, and the temperature was maintained at 23 ± 3 °C with a relative humidity of 55 ± 15%. All animals were used in the experiments only after acclimatization for at least 1 week.

### 2.4. Venous Occlusion Procedures

The epigastric vein of a pig, which was the site of administration of the test substance, travels along the abdominal wall and enters the abdominus rectus vein near the rib. The right epigastric vein (as the treatment site) [23] and the left epigastric vein without an adhesive were used as the control group (Figure S2).

After a pig was placed in the supine position, the abdomen was sterilized and covered with a disinfectant cloth. After the epigastric vein was located within the abdomen using an ultrasonography system (GE Healthcare, Chicago, IL, USA), the vein was punctured using a micropuncture set through a standard puncture method, and a guide wire was inserted. The introducer was inserted through the path of the inserted guide wire. After the adhesive was transferred to a 5 mL syringe, a 5 Fr catheter was combined with the syringe. The guide wires were removed after the introducer was inserted into the epigastric vein. The 5 Fr catheter was placed in the target vein under ultrasound observation of the epigastric vein through an introducer. After the injection location was determined, 0.1 mL of adhesive was injected, while the end of the treatment area where the catheter was placed was pressed using an ultrasound probe. Following the first injection, the catheter was moved back 1 cm, and a second injection was performed. The catheter was then pulled back 3 cm, and the injection site was pressed for 3 min for vascular embolization. Next, injections were performed at 3 cm intervals, and each injection site was pressed for 30 s. The final injection point was close to the puncture site (3 cm or less). After the injections were completed, the catheter and introducer were consecutively removed. Each puncture site was pressed with a hand to stop bleeding and then sutured using 4-0 monofilament nylon (Ethilon^®^, Ethicon-Johnson & Johnson, Mumbai, India). On the day of the procedure (1 d) and at 7 and 14 d following the procedure, an ultrasound was used to check for venous occlusion at the treatment site. The presence and movement of the injected adhesive were monitored to determine whether the vein was occluded. Fourteen days after the procedure, an ultrasound was used to confirm the presence and movement of the adhesive at the treatment site, and an autopsy was performed (Figure S3). The right epigastric vein was harvested, and the tissue was collected and stained. A biopsy was performed to determine the degree of inflammation.

### 2.5. Measurement Procedures 

For the procedure, we studied the embolic efficacy and intravascular distribution of the injected adhesive, migration of the adhesive, and immune reaction at the injected site. Follow-up ultrasound imaging was also performed 7 and 14 d after EGpresto injection as compared with the ultrasound imaging before injection. After the follow-up ultrasound, the mini-pigs were sacrificed on Day 14, and the left and right epigastric veins were surgically removed for histopathological examination. 

### 2.6. Histological Examination

To evaluate the immune reaction of EGpresto at the injected site, histopathological studies were performed. Fragments of both epigastric veins (average length of approximately 30 cm) were taken and processed in 10% formalin, dehydrated using ethanol, sheared in xylene, and fixed in paraffin blocks. The fragments were then stained with hematoxylin-eosin (H&E) [24]. To examine the histopathological changes, optical microscopic analyses were conducted to determine the following: the pattern of the test substance that had been injected into the vein, the surrounding tissues of inflammatory cells such as macrophages and neutrophils, the presence of invasion in the blood vessel wall and the periphery of the test substance in the vein, necrosis of the surrounding tissues, and apoptosis.

## 3. Results and Discussion

### 3.1. Viscosity of Two Adhesives

**Figure 2:**
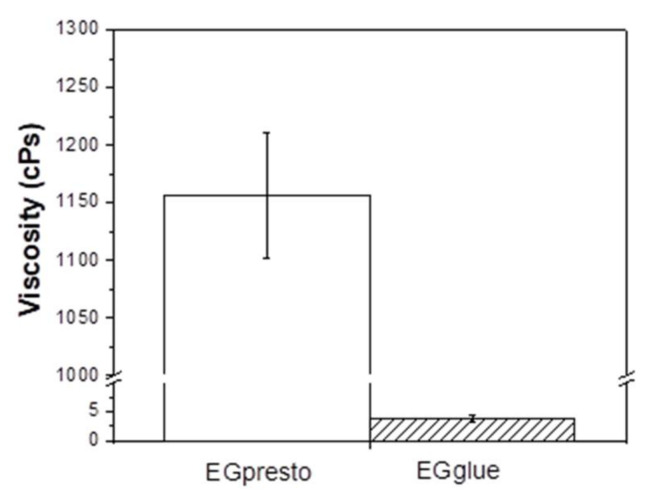
Viscosities of EGglue and EGpresto measured at 25 °C. The respective viscosity values were averaged from three independent measurements, and the calculated results were 3.7 ± 0.6 cPs for EGglue and 1150 ± 55 cPs for EGpresto (*p* < 0.005). Statistical analysis method: Student’s *t*-test.

The viscosities of EGglue and EGpresto were measured at 25 °C using a cone-plate viscometer. As Figure 2 shows, whereas the apparent viscosity for pure NBCA glue was 3.7 ± 0.6 cPs, EGpresto showed a fairly high viscosity of 1150 ± 55 cPs due to the presence of the polymer additives. 

From the Statistical analysis on the viscosity data of EG presto and EG glue based on Student’s *t*-test method, *p*-value less than 0.005 was obtained implying two data sets are significantly different.

### 3.2. Heat of Polymerization and Curing Time

**Figure 3:**
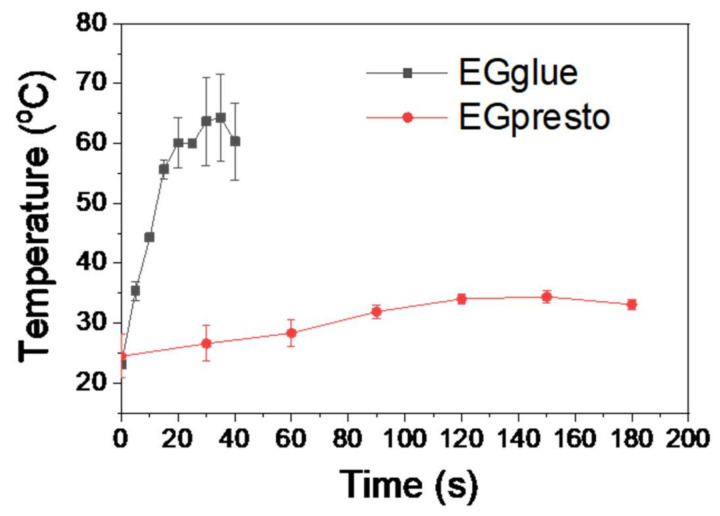
Temperature changes upon polymerizations of EGglue and EGpresto for measuring heat of polymerization and curing time. Temperatures from three independent measurements were averaged. The heat of polymerization for EGglue and EGpresto was measured to be 64 ± 7 °C and 34 ± 1 °C, respectively. The polymerization times were approximately 30 s and 120–150 s for EGglue and EGpresto, respectively.

Figure 3 shows the temperature changes of EGglue and EGpresto when the same amount of each adhesive was mixed with an aqueous culture medium. Note that in the test conditions, we needed to mimic the conditions of the human body. The curing time of NBCA can be changed depending on the test method and materials used, such as deionized water, normal saline solution (0.9% NaCl solution), dextrose solution (0.5%), and cell culture media and simulated body fluid [25]. We adopted the modified Eagle’s medium because it is widely used for mammalian cell cultures. We also used an extraction solution for biological safety tests based on ISO-10993-5 [26]. The heat of polymerization is defined as the time required for the system to reach its maximum temperature during the polymerization reaction of NBCA. From three independent measurements, the average heat of polymerization for EGglue was observed to be 64 ± 7 °C, whereas that of EGpresto was measured to be 34 ± 1 °C. It was clear that the heat of polymerization for the highly viscous NBCA glue decreased to 53% of the value of a low-viscosity NBCA. In terms of temperature change (ΔT; maximum temperature–initial temperature), the difference between the two glues was more obvious, where EGpresto exhibited a ΔT of only 9 °C as compared to that of EGglue of 39 °C. The decreased heat of polymerization for EGpresto was related to the retarded polymerization time, because the produced heat was dissipated to the surroundings during polymerization. The polymerization time for EGglue was measured to be approximately 30 s, whereas that of EGpresto ranged from 120 to 150 s, as shown in Figure 3. 

Retardation of polymerization time in EGpresto can mainly be attributed to the hindered diffusion of NBCA molecules within the viscous medium, as predicted by the Stokes–Einstein equation for diffusion [27].

D = k_B_T/6πηr

where D, k_B_, η, and r denote the diffusion coefficient, Boltzmann constant, viscosity, and hydrodynamic radius of the molecule, respectively.

As the diffusion of the NBCA monomer slows down, the propagation rate of polyNBCA also decreases, resulting in a lowered heat of polymerization, as shown in Figure 3. The results confirmed a much lower heat-burn problem from NBCA polymerization during the vascular occlusion procedure.

### 3.3. Examination of Vascular Occlusion

**Figure 4:**
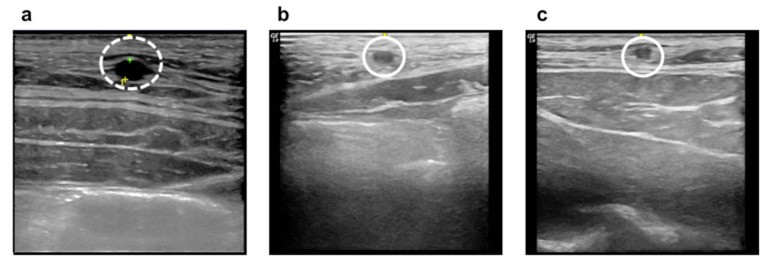
Ultrasound images of medical adhesive (EGpresto) injection sites: (**a**) normal epigastric vein before the procedure (dashed circle), (**b**) 7 d after injection, and (**c**) 14 d after injection (solid circles).

A vascular occlusion test for mini-pigs was conducted to confirm the efficacy and safety of a high-viscosity adhesive. Migration of the adhesive to the lungs has been reported as a side effect of NBCA-based liquid embolic agents during blood vessel occlusion, causing pulmonary embolization [28,29,30]. In a recent case study on VenaSeal^®^ (Medtronic, Fridley, MN, USA), researchers investigated whether migration of the adhesive occurs within a vessel of a pig within a period of 14 d. In this study, we examined the migration of the injected adhesives 7 and 14 d following the procedure. The average diameter of the epigastric veins of the three mini-pigs before the procedure was measured to be 3.1 ± 0.3 mm using an ultrasonographic system. The ultrasound results 7 d prior to and 14 d after the procedure confirmed that all three animals showed a complete postoperative vein-occlusion rate as compared with the untreated vein before the procedure (Figure 4). All three pigs maintained healthy conditions throughout the examination period following the embolization procedures.

**Figure 5:**
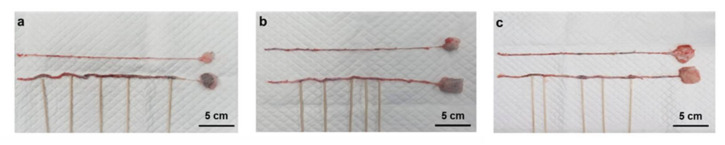
(**a**–**c**) Optical images of the EGpresto-injected epigastric veins (below) and non-treated veins (above) exposed from three mini-pigs after 14 d of the procedure. The injected sites for the treated veins are indicated by wooden sticks.

For the sacrificed animals, the left and right epigastric veins (operated and normal blood vessels) were exposed, and the locations of the injected adhesive were checked and indicated by sticks after removal, as shown in Figure 5.

It was confirmed that the EGpresto-treated vessels were well occluded in all three animals. As shown in the extracted epigastric veins in Figure 5, the injected adhesive could be visualized at the occlusion site without migration. The intervals of the injected adhesives in the treated vessels could also be clearly recognized for each mini-pig, whereas all the non-treated veins appeared clean without nodules. 

### 3.4. Histological Study

**Figure 6:**
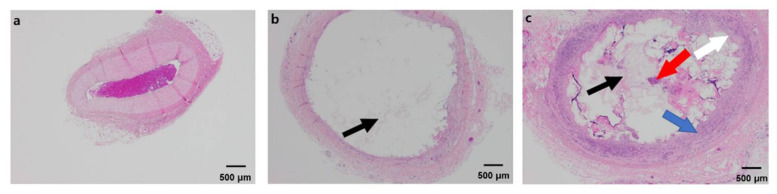
Histological images of EGpresto stained by H&E (X40). (**a**) normal veins (control), (**b**,**c**) two different spots from EGpresto-injected veins. In (**a**), a control epigastric vein image shows typical tissue layers and normal intima, media, and adventitia layers. Only a blood clot appeared in the endovascular area. This control epigastric vein sample was harvested from the same pig as in (**b**,**c**) but at an opposite side. In (**b**,**c**), the adhesive materials are indicated by black arrows. The red arrow in (**c**) indicates an inflammatory cell. Blue and white arrows indicate a fibrosis area and intima proliferation, respectively. However, the inflammatory cells are defined only in (**c**). These epigastric vein samples were harvested from a different injection site of the same epigastric vein.

From the histological changes visualized by optical microscopy after H&E staining, it was confirmed that the EGpresto-treated veins contained the experimental substances inside the blood vessel 14 d after the procedure (Figure 6). Because the tissue occupied more than half the lumen size (approximately 3.1 mm), several small calcifications formed during the occlusion period. 

Figure 6 shows the histological changes in the normal and injected veins at the occlusion site. Foreign-body reactions were observed in the treated vessel, and acute and moderate chronic inflammation and eosinophilic infiltration were identified on the vessel wall. In addition, no test material visibly leaked out of the blood vessels (Figure 6b,c). Figure 6b shows that at some of the EGpresto injected sites, there was only adhesive material without visible foreign-body reaction. However, as shown in Figure 6c, inflammatory cells were found around the adhesive material and designated fibrosis area, and intima proliferation occurred. However, the injected adhesive was not found in other sites such as the inner or outer vein walls. In addition, intima proliferation and the fibrosis area were limited to the intravascular site without other sites. Immune reactions were defined just near the vicinity of the material-injected area; these reactions seemed to be the only foreign-body reaction (and were not severe). From the histological images in Figure 6b,c, no necrosis or apoptosis were observed on the surrounding tissues. Therefore, EGpresto was confirmed to be a non-toxic and safe embolic agent. This is a promising result for the developed adhesive, indicating that it can be effectively used for medical applications, including as a liquid embolic agent for transcatheter arterial embolization and arteriovenous malformations and in treating varicose veins.

## 4. Conclusions

We investigated the physical properties of EGpresto, a new NBCA-based adhesive recently developed for venous embolization. As EGpresto has optimized viscosity for controlled polymerization time and heat of polymerization, significantly different physical properties were observed in the study in comparison with EGglue, which is a low-viscosity NBCA glue. The heat of polymerization value of EGpresto was found to be lower than body temperature, whereas that of the low-viscosity NBCA glue was measured to be 64 °C. In addition, the polymerization time of EGpresto was delayed by more than 2 min as compared with that of EGglue. In an animal study using three mini-pigs, intravenous injection of EGpresto was conducted, and only moderate immune reactions were observed through histological analysis. The results confirmed the safety and efficacy of EGpresto as a venous closure system in an animal model, thus promising effective utilization of the cyanoacrylate ablation device in disease models such as varicose veins.

## Data Availability

Not applicable.

## Supplementary Materials

The following are available online at https://www.mdpi.com/article/10.3390/ma14133527/s1, Figure S1: Image of the full device set of EGpresto, a highly viscous N-butyl cyanoacrylate based medical device set, Figure S2: Photograph showing an injection of EGpresto into a right epigastric vein of a pig, Figure S3: Optical images of the EGpresto-injected epigastric veins exposed from a sacrificed pig.
