# Supplementary material for: In Vivo Animal Study of a Highly Viscous N-butyl Cyanoacrylate Medical Adhesive for Intravenous Embolization
Source: Materials (Basel). 2021 Jun 24;14(13):3527. doi: 10.3390/ma14133527 (PMC8269526; doi:10.3390/ma14133527)
Supplement: Supplementary file 1 [file materials-14-03527-s001.zip › materials-1240562-supplementary.pdf]

materials

Supplementary Materials

In Vivo Animal Study of a Highly Viscous N-butyl Cyanoacrylate Medical Adhesive for Intravenous Embolization

Jae-Won Seo[1], Habeen Park[1,2], Dogeun Kim[1], Seoyun Lee[1], Young Gook Koh[1], Jang Yong Kim[3], Insoo Park[4] and Wonmok Lee[1,2,*]

1 ENGAIN Co. Ltd., 700, Daewangpangyo-ro, Bundang-gu, Seongnam-si, Gyeonggi-do 13488, Korea; jwseo@engain.co.kr (J.-W.S.); clooney.park@engain.co.kr (H.P.); dgkim@engain.co.kr (D.K.); sylee@engain.co.kr (S.L.); young.koh@engain.co.kr (Y.G.K.)

2 Department of Chemistry, Sejong University, 209, Neungdong-ro, Gwangjin-gu, Seoul 05006, Korea

3 Department of Surgery, Seoul St. Mary's Hospital, College of Medicine, The Catholic University of Korea, Seoul 06591, Korea; vascularkim@catholic.ac.kr

4 Charm Vascular Clinic, 1814 Nambu-sunhwan-ro, Gwanak-gu, Seoul 08787, Korea; insoo.park.md@gmail.com

* Correspondence: wonmoklee@sejong.ac.kr; Tel.: +82-10-4914-3598

Citation: Seo, J.-W.; Park, H.; Kim, D.; Lee, S.; Koh, Y.G.; Kim, J.Y.; Park, I.; Lee, W. In vivo animal study of a highly viscous N-butyl cyanoacrylate medical adhesive for intravenous embolization. *Materials* **2021**, *14*, 3527. https://doi.org/10.3390/ma14133527

Academic Editor: José Luis Gómez Ribelles

Received: 14 May 2021
Accepted: 17 June 2021
Published: 24 June 2021

Publisher's Note: MDPI stays neutral with regard to jurisdictional claims in published maps and institutional affiliations.

Copyright: © 2021 by the authors. Licensee MDPI, Basel, Switzerland. This article is an open access article distributed under the terms and conditions of the Creative Commons Attribution (CC BY) license (http://creativecommons.org/licenses/by/4.0/).

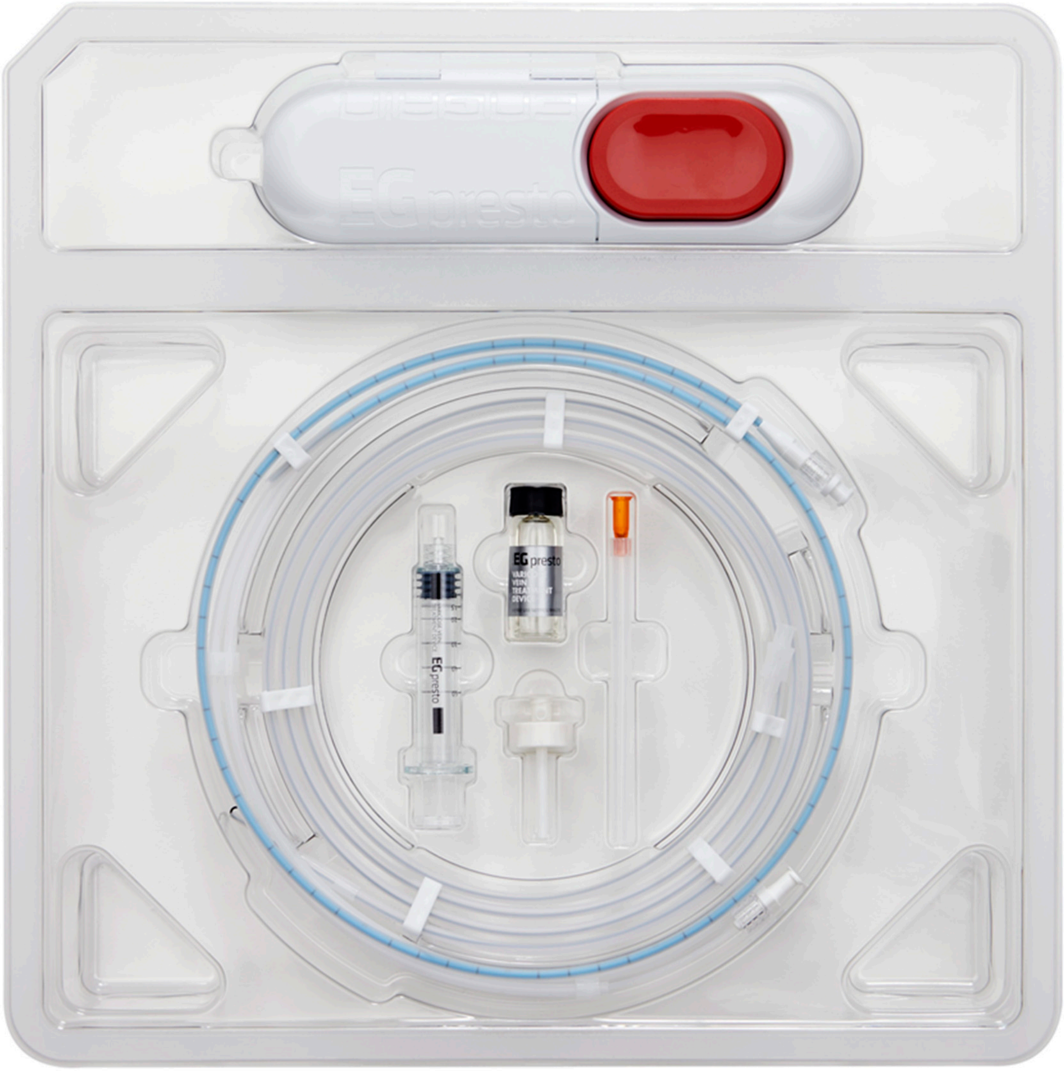

Figure S1. Image of the full device set of EGpresto, a highly viscous N-butyl cyanoacrylate based medical adhesive. An EGpresto device set contains highly viscous glue in a vial, injector, guide wire, introducer & catheter , 16-G needle, vial connector, and a 5-cc syringe.

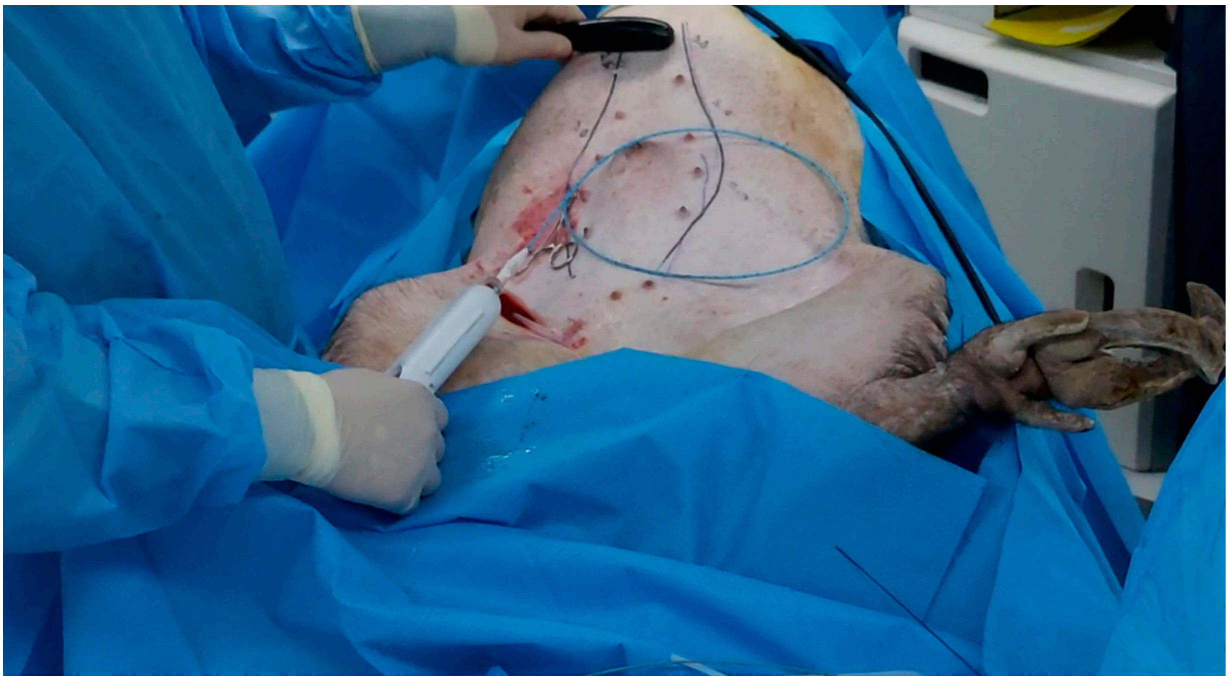

Figure S2. Photograph showing an injection of EGpresto into a right epigastric vein of a mini-pig. The catheter position and adhesive injection were confirmed under ultrasound system.

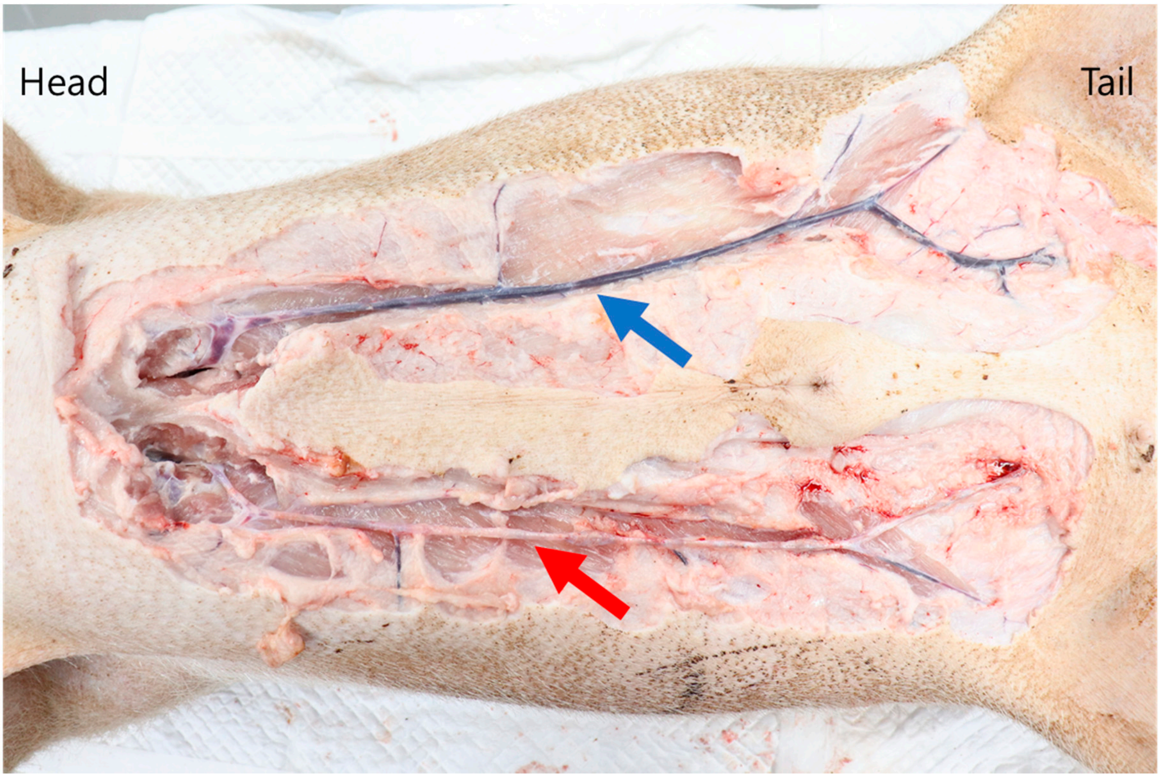

Figure S3. Optical images of the EGpresto-injected epigastric vein (red arrow) and non-treated vein (blue arrow) exposed from a sacrificed mini-pig after 14 d of the procedure. The color of the non-treated vein appears dark blue (normal vein color) while the treated vein is significantly different because the blood flow in the treated vein was blocked by adhesive material.
